# Military Prophylaxis of Venereal Diseases

**Published:** 1918-10

**Authors:** 


**A Monthly Review of Medicine and Surgery**

EDITOR AND PUBLISHER, DR. A. L. BENEDICT, 228 Summer St., corner of Elmwood Ave., Buffalo, (Address for all communications. Please make personal and telephone calls before 1 P. M.)

Third, in age, on the western continent. Independent, but supports professional organizations. News limited mainly to Western N. Y. and Penn.

Subscription $2.00 a year; $3.00 for two years in advance. Changes and errors in address or failure or duplication of delivery, should be reported immediately.

Yearly Volume 74 OCTOBER, 1918 Number 3

### Military Prophylaxis of Venereal Diseases.

Both in the Navy and the Army of the U. S., there has been adopted as a basic principle of preventing venereal diseases, not a method of abortive treatment misnamed prophylaxis, not a system of inspection which cannot either practically or theoretically diagnose and quarantine before the development of an infection, but the simple, efficient and radical moral means. So far as we know, this is the first time in military history in which this method has been adopted as a general policy by the military authorities of any nation and in which the machinery of military discipline has been employed on a large scale and in good faith for this purpose.

2,979 years ago, a general of good repute, even today, in commandeering food found himself up against this problem: The only available supply was the sacred bread of a small temple—or at least what other nations would so call it. The priest in charge hesitated but intimated that the military exigencies would justify the use of this supply, if, at least, the men had kept themselves from women. The general replied with pride that there had been no women available for about three days "since I came out" and this prolonged continence was satisfactory to the priest. (See I Samuel, Chapter 21, part of the narrative of David). The contrast with the modern ideal is striking and neither David nor other generals of history have considered prolonged continence on a large scale possible.

It is not the entire purpose of this article to show how much superior our military morals are to those of the past nor to those of some nations at present. Some have explained certain atrocities, marked by non-professional mastectomy as due to the prohibition of soldiers already infected with venereal diseases, from applying to regularly licensed female camp followers, or even of leaving their own victims capable of conveying disease to comrades of the same army. What we wish to emphasize is the tremendous difficulty of effectively carrying out in practice, the theoretically ideal means of venereal prophylaxis to which we are committed. This is shown by previous military experience for centuries, by a knowledge of human frailty which it is the merest hypocrisy to pretend that we do not possess, by current statistics which show that the ideal method has failed within a very brief period, so brief as to warrant considerable scepticism of success after the lapse of a sufficient time to allow the development of severe sexual hunger. Expressed arithmetically, the problem is to reduce an existing and fairly well established annual rate of somewhere between 50 and 150, not to include extreme limits in either direction, to zero, in a total of 2,000,000 according to present estimates of our military force in the near future. We have purposely stated the problem in abstract rather than concrete numbers because many persons consider it an indication of personal probity to express views regarding the venereal problem which they would not think of doing with regard to any other. Such a course is neither scientific nor ultimately moral for the simple reason that it is not truthful. Suppose, for example, we conceded that the 100 grams of fat that most persons ingest daily could perfectly well be reduced to zero—and there is a good scientific argument for this contention. This does not imply that 2,000,000 persons will voluntarily subject themselves to a fat-free ration. The experience of Germany with regard to fats well illustrates the difficulty of the problem.

Suppose that the method of venereal prophylaxis to which we are committed does fail—or rather, in the light of actual adverse reports from encampments under far more favorable conditions than can be maintained merely on account of the effects of lapse of time alone—let us be honest enough to say: suppose this method keeps on failing, what are we going to do? Are we going to act as in the case of typhoid, use all possible methods of prophylaxis and then of therapeutics in case of ultimate failure or are we going to stake everything on the one ideal method?

